# Supplementary material for: Ecological niche modeling reveals habitat differentiation and climatic vulnerability in two imperiled, sympatric southern Appalachian carnivorous plants
Source: Am J Bot. 2026 Apr 23;113(5):e70194. doi: 10.1002/ajb2.70194 (PMC13206205; doi:10.1002/ajb2.70194)
Supplement: Supplementary file 1 — Appendix S1. Table of bioclimatic variables considered for niche modeling. [file AJB2-113-e70194-s001.docx]

**Appendix S1.** Table listing all bioclimatic variables considered for niche modeling.

| Variable Code | Variable Name |
| --- | --- |
| BIO01 | Annual mean temperature |
| BIO02 | Mean diurnal range (mean of monthly(max-min temperature)) |
| BIO03 | Isothermality (BIO02/BIO07) |
| BIO04 | Temperature seasonality (SD x 100) |
| BIO05 | Maximum temperature of the warmest month |
| BIO06 | Minimum temperature of the coldest month |
| BIO07 | Annual temperature range (BIO05-BIO06) |
| BIO08 | Mean temperature of the wettest quarter |
| BIO09 | Mean temperature of the driest quarter |
| BIO10 | Mean temperature of the warmest quarter |
| BIO11 | Mean temperature of the coldest quarter |
| BIO12 | Annual precipitation |
| BIO13 | Precipitation of the wettest month |
| BIO14 | Precipitation of the driest month |
| BIO15 | Precipitation seasonality (coefficient of variation) |
| BIO16 | Precipitation of the wettest quarter |
| BIO17 | Precipitation of the driest quarter |
| BIO18 | Precipitation of the warmest quarter |
| BIO19 | Precipitation of the coldest quarter |
