# Supplementary material for: Ecological niche modeling reveals habitat differentiation and climatic vulnerability in two imperiled, sympatric southern Appalachian carnivorous plants
Source: Am J Bot. 2026 Apr 23;113(5):e70194. doi: 10.1002/ajb2.70194 (PMC13206205; doi:10.1002/ajb2.70194)
Supplement: Supplementary file 2 — Appendix S2. Contemporary suitability scores for sites containing either S. rubra subsp. jonesii or S. purpurea var. montana. [file AJB2-113-e70194-s003.docx]

**Appendix S2.** Contemporary suitability scores for sites containing either *S. rubra* subsp. *jonesii* or *S. purpurea* var. *montana*.
