# Supplementary material for: Ecological niche modeling reveals habitat differentiation and climatic vulnerability in two imperiled, sympatric southern Appalachian carnivorous plants
Source: Am J Bot. 2026 Apr 23;113(5):e70194. doi: 10.1002/ajb2.70194 (PMC13206205; doi:10.1002/ajb2.70194)
Supplement: Supplementary file 3 — Appendix S3. Suitability maps for Sarracenia rubra subsp. jonesii (SRJ) and Sarracenia purpurea var. montana (SPM) at near‐present historical and three future time horizons (2021–2040, 2041–2060, 2061–2080) under the most likely emissions reduction scenario (SSP 370). [file AJB2-113-e70194-s004.docx]

**Appendix S3.** Suitability maps for *Sarracenia rubra* ssp. *jonesii* (SRJ) and *Sarracenia purpurea* var. *montana* (SPM) at present and three future time horizons (2021-2040, 2041-2060, 2061-2080) under SSP 370. Darker colors indicate areas with suitability scores > 0.6, while lighter shaded areas with suitability scores < 0.6 and > 0.4. Black outlines show the actual range extent for each taxon.
