# Supplementary material for: Ecological niche modeling reveals habitat differentiation and climatic vulnerability in two imperiled, sympatric southern Appalachian carnivorous plants
Source: Am J Bot. 2026 Apr 23;113(5):e70194. doi: 10.1002/ajb2.70194 (PMC13206205; doi:10.1002/ajb2.70194)
Supplement: Supplementary file 4 — Appendix S4. Projected estimates for area of habitat of each taxon across all time horizons and SSPs, for areas with a suitability score >0.4. [file AJB2-113-e70194-s002.docx]

**Appendix S4.** Projected estimates for area of habitat of each taxon across all time horizons and SSPs, for areas with a suitability scores >0.4.
